# Higher cholesterol levels, not statin use, are associated with a lower risk of hepatocellular carcinoma

**DOI:** 10.1038/s41416-019-0691-3

**Published:** 2019-12-20

**Authors:** Sang-Wook Yi, Se Hwa Kim, Ki Jun Han, Jee-Jeon Yi, Heechoul Ohrr

**Affiliations:** 1 Department of Preventive Medicine and Public Health, Catholic Kwandong University College of Medicine, Gangneung, 25601 Republic of Korea; 2 Department of Internal Medicine, Catholic Kwandong University College of Medicine, International St. Mary’s Hospital, Incheon, 22711 Republic of Korea; 3 Hepatobilliary Center, Catholic Kwandong University College of Medicine, International St. Mary’s Hospital, Incheon, 22711 Republic of Korea; 4 Institute for Occupational and Environmental Health, Catholic Kwandong University, Gangneung, 25601 Republic of Korea; 5 Department of Preventive Medicine, Yonsei University College of Medicine, Seoul, 03722 Republic of Korea

**Keywords:** Risk factors, Hepatocellular carcinoma, Predictive markers

## Abstract

We aimed to examine whether statin users have a lower risk of hepatocellular carcinoma (HCC) after careful consideration of prevalent statin use and cholesterol levels. During a mean prospective follow-up of 8.4 years in 400,318 Koreans, 1686 individuals were diagnosed with HCC. When prevalent users were included, HCC risk was reduced by >50% in statin users, regardless of adjustment for total cholesterol (TC). When prevalent users were excluded, new users who initiated statins within 6 months after baseline had a 40% lower risk of HCC (hazard ratio [HR] = 0.59) in a TC-unadjusted analysis. However, this relationship disappeared (HR = 1.16, 95% CI = 0.80–1.69) after adjusting for TC levels measured within 6 months before statin initiation. TC levels had strong inverse associations with HCC in each model. High cholesterol levels at statin initiation, not statin use, were associated with reduced risk of HCC. Our study suggests no protective effect of statins against HCC.

## Background

Over 20 observational studies have suggested that statins protect against hepatocellular carcinoma (HCC) development.^1–5^ Most of those studies included individuals who had been using statins for some time before enrolment, although the inclusion of prevalent users can introduce biases in observational studies of efficacy.^6^ Hypercholesterolemia is the main indication for statin use. Blood cholesterol levels have been reported to have inverse associations with HCC in several studies.^7^ However, few studies of statin efficacy have controlled for cholesterol concentrations.^8^ Our prospective cohort study aimed to examine whether statin use was associated with a reduced risk of HCC after careful consideration of cholesterol levels and exclusion of prevalent users.

## Methods

The main study cohort (*n* = 400,318), comprising participants in 2004–2007 health examinations administered through the National Health Insurance Service (NHIS) of Korea (Fig. S1), was followed-up until December 31, 2013 for HCC incidence via record linkage to hospital discharge records.^9^ Hazard ratios (HRs) for HCC incidence were calculated using Cox proportional hazards models after adjustment for multiple confounders (see Table 1 footnote). Information on statin use (atorvastatin, fluvastatin, lovastatin, pitavastatin, pravastatin, rosuvastatin, and simvastatin) was collected from the NHIS prescription database. Defined daily doses (DDDs) were used to quantify statin usage. Total cholesterol (TC), glucose, and alanine aminotransferase were assayed using fasting serum samples. Body mass index was measured. Smoking status, alcohol use, physical activity, and history of cancer were assessed via a questionnaire in previous (2002–2003) health examinations. More details on the study methods can be found in the supplemental text.

**Table 1:** HRs^a^ for HCC incidence associated with statin use and total cholesterol (TC) in main cohort participants (*n* = 400,318)

			Multivariable-adjusted (except TC)		Multivariable-adjusted (including TC) (main analysis)		Analysis when only those who initiated statins within 1 year after baseline were considered as users		
TC or statin use characteristics	No. of participants	HCC cases	*p*-value	HR (95% CI)	*p*-value	HR (95% CI)	HCC cases	*p*-value	HR (95% CI)
1 mmol/L (39 mg/dL) increase in TC^b^	400,318	1686			<0.001	0.54 (0.51–0.58)			
*Statin use within 6 months after baseline*^c^									
No use	389,052	1657		1.00 (Reference)		1.00 (Reference)			
Use	11,266	29	0.005	0.59 (0.41–0.85)	0.427	1.16 (0.80–1.69)			
≤91 cDDDs	9350	24	0.007	0.57 (0.38–0.86)	0.597	1.12 (0.74–1.68)			
>91 cDDDs	1916	5	0.395	0.68 (0.28–1.64)	0.402	1.46 (0.60–3.52)			
No use	389,052	1657		1.00 (Reference)		1.00 (Reference)			
<40 cDDDs (<50th percentile)	4812^d^	17	0.266	0.76 (0.47–1.23)	0.144	1.43 (0.88–2.32)			
≥40 cDDDs (≥50th percentile)	6454^d^	12	0.006	0.45 (0.25–0.79)	0.769	0.92 (0.52–1.63)			
Per 60 cDDD increase	400,318	1686	0.020	0.64 (0.44–0.93)	0.633	1.08 (0.78–1.49)			
1 mmol/L (39 mg/dL) increase in TC^e^	400,318	1686			<0.001	0.55 (0.52–0.59)	1686	<0.001	0.55 (0.52–0.58)
*Statin use within 2 years after baseline*^c^									
No use	366,896	1620		1.00 (Reference)		1.00 (Reference)	1642		1.00 (Reference)
Use	33,422	66	<0.001	0.44 (0.34–0.57)	0.004	0.69 (0.54–0.89)	44	0.669	0.94 (0.69–1.27)
≤182 cDDDs	25,288	51	<0.001	0.46 (0.34–0.60)	0.016	0.71 (0.53–0.94)	31	0.839	1.04 (0.72–1.49)
183–365 cDDDs	5741	8	<0.001	0.30 (0.15–0.61)	0.042	0.48 (0.24–0.97)	7	0.195	0.61 (0.29–1.29)
>365 cDDDs	2393	7	0.228	0.63 (0.30–1.33)	0.819	1.09 (0.52–2.30)	6	0.903	1.05 (0.47–2.35)
No use	366,896	1620		1.00 (Reference)		1.00 (Reference)	1642		1.00 (Reference)
<30 cDDDs (1st quartile)	7850	23	0.042	0.65 (0.43–0.99)	0.954	0.99 (0.65–1.49)	14	0.205	1.41 (0.83–2.39)
30–79 cDDDs (2nd quartile)	8626	12	<0.001	0.32 (0.18–0.57)	0.015	0.49 (0.28–0.87)	6	0.291	0.65 (0.29–1.45)
80–179 cDDDs (3rd quartile)	8322	15	<0.001	0.40 (0.24–0.66)	0.092	0.64 (0.39–1.07)	10	0.947	0.98 (0.52–1.83)
≥180 cDDDs (4th quartile)	8558	16	<0.001	0.40 (0.25–0.66)	0.100	0.66 (0.40–1.08)	14	0.394	0.79 (0.47–1.35)
Per 60 cDDD increase	400,318	1686	<0.001	0.80 (0.73–0.88)	0.055	0.92 (0.84–1.00)	1686	0.403	0.96 (0.89–1.05)
No use	366,896	1620		1.00 (Reference)		1.00 (Reference)			
Statin initiation≤182 days after baseline	11,266	29	0.002	0.56 (0.39–0.81)	0.563	1.12 (0.77–1.62)			
Statin initiation 183–365 days after baseline	7085	15	0.002	0.45 (0.27–0.76)	0.125	0.67 (0.40–1.12)			
Statin initiation 366–730 days after baseline	15,071	22	<0.001	0.34 (0.22–0.52)	<0.001	0.47 (0.31–0.72)			

To convert cholesterol from mg/dL to mmol/L, multiply by 0.02586

*cDDDs* cumulative DDDs; *CI* confidence interval; *DDD* defined daily dose; *HCC* hepatocellular carcinoma; *HR* hazard ratio

^a^HRs were calculated by Cox models stratified by age (baseline age, years: 40–44, 45–54, 55–64, 65–74, ≥75), after adjustment for age at baseline, sex, pre-existing diabetes, smoking status, alcohol use, physical activity, hepatitis B virus infection, hepatitis C virus infection, liver cirrhosis, body mass index, alanine aminotransferase levels, and total cholesterol (when applicable)

^b^Statin use within 6 months after baseline was adjusted for in the multivariable analysis

^c^The first 6 months of follow-up were excluded

^d^Because 833 users were prescribed exactly 40 cDDDs, during 6 months after baseline health examination, the number of participants were different between two groups

^e^Statin use within 2 years after baseline was adjusted for in the multivariable analysis

## Results

**Fig. 1:**
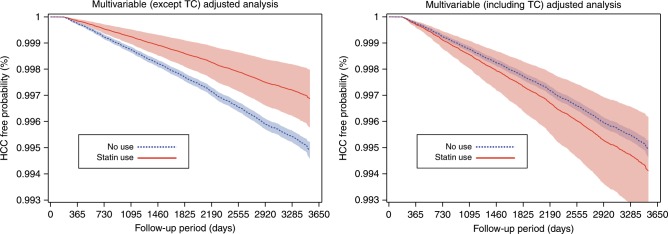
Multivariable-adjusted hepatocellular carcinoma (HCC)-free probability curves according to statin use within 6 months after baseline health examination. The first 6 months of follow-up were excluded to minimise immortal time bias. HCC-free probability and 95% confidence intervals were calculated using Cox proportional hazard models after adjustment for age at baseline, sex, pre-existing diabetes, smoking status, alcohol use, physical activity, hepatitis B virus infection, hepatitis C virus infection, liver cirrhosis, body mass index, alanine aminotransferase levels, and total cholesterol levels (when applicable).

During 8.4 mean years of follow-up, 1686 individuals were diagnosed with HCC. They were more likely to be statin non-users than persons without HCC (Table S1). Statin users tended to be older, female, never-smokers, and high-income earners and to have higher TC levels, diabetes, and less liver disease than non-users (Table S2). In the analysis that included prevalent users (Table S3), the risk of HCC was generally reduced by >50% in statin users, regardless of dosage and adjustment for TC. When prevalent users were excluded, new users who initiated statins within 6 months after baseline had a 40% lower risk of HCC (HR = 0.59) in a TC-unadjusted analysis. However, this relationship disappeared (HR = 1.16, 95% CI = 0.80–1.69) after adjusting for baseline TC levels measured within 6 months before statin initiation (Table 1, Fig. 1). Additionally, when stratified analyses by TC levels were done, new users did not have a lowered risk of HCC in any TC group (Table S4)

.

Interestingly, statin use within 2 years after baseline was associated with an ~30% lower risk of HCC than non-use in the multivariable-adjusted analysis (HR = 0.69, Table 1). In dose-risk analyses, the low- to moderate-dosage groups of ≤182 (HR = 0.71) and 183–365 (HR = 0.48) cDDDs within 2 years had lower risks, but not the highest dosage group of >365 cDDDs (HR = 1.09). The sensitivity analyses that excluded statin users within the past 2 years to minimise immortal time bias showed similar results (Table S5). When the timing of statin initiation was considered, statin initiation 1 year after baseline (HR = 0.47), but not within 6 months after baseline (HR = 1.12), was associated with lowered risk (Table 1). When only those who initiated statins within 1 year after baseline were considered as users, statin users (HR = 0.94) and each dosage group within 2 years after baseline had no lowered risk. Meanwhile, strong inverse associations with TC were found in each model.

## Discussion

In the analysis that included prevalent users, statin users showed a >50% risk reduction for HCC. When prevalent users were excluded, new users also had a lower risk of HCC in a TC-unadjusted analysis. However, after adjustment for baseline TC levels measured within 6 months before statin initiation, the protective effects of statins against HCC disappeared. Blood TC levels had a strong inverse association with HCC incidence.

A major bias related to prevalent users is the inability to control for confounders that may be altered by statins.^10^ Confounding by indication is another source of bias. When associations between statin use and HCC risk are examined, the general indication for statins (i.e., cholesterol levels) is a confounder, since cholesterol levels have been inversely associated with HCC.^7^ Therefore, cholesterol levels are a key contributor both to prevalent user bias and to confounding by indication. Surprisingly, few studies on the effects of statin use on HCC have carefully accounted for cholesterol levels and excluded prevalent users.^8^ A few recent studies applied new-user designs, but, unfortunately, cholesterol levels were not adjusted for or most cholesterol measurements were made after statin use.^3,5^ However, our study demonstrated that adjustment for cholesterol levels cannot eliminate bias, especially when prevalent users or cholesterol levels measured after statin initiation were included in the analysis, and that measurements made 0.5–1 year before statin initiation may not reflect the values at the time of the decision to initiate statins. Meanwhile, it is possible that physicians avoid prescribing statins for individuals with liver disease. However, after adjustment for statin-unaffected TC, new users had no beneficial effect regardless of liver disease status (Table S6). A secondary analysis of randomised trials of statins on cardiovascular outcomes, which are free from these biases, showed that statins had no protective effects against HCC development (incidence rate ratio = 1.06), although this finding was based on a small number of cases (*n* = 68).^11^

Our finding that later initiation or recent use of statins was associated with lower risk are in accordance with the findings of a UK study that recent users, but not past users, had lower risks of HCC.^4^ Immortal time bias may partly, but not entirely, explain this result, since excluding the first 2 years of follow-up modestly changed the findings. The most recent statin users may have a lowered risk, probably not because of statin use, but because of their recent high cholesterol levels, which were not prominent at baseline; since lower cholesterol levels are a marker of liver disease and its severity,^12^ cholesterol levels (the indication for statins) have strong inverse associations with HCC.^7^ Analyses of cholesterol levels in the 2002–2003 and 2004–2007 health examinations showed that new initiators had higher levels than current non-users (intergroup difference; groups 5–8 vs. groups 1–4 in 2002–2003; groups 2, 6, 10, 14 vs groups 1, 5, 9, 13 in 2004–2007) and in recent measurements than in previous measurements (intragroup difference; group 2 in 2004–2007 vs. in 2002–2003), while those who discontinued statins had lower levels in recent measurements than in previous measurements (intragroup difference; group 5 in 2004–2007 vs. in 2002–2003) (Table S7).

Previous studies have reported non-linear associations of statin dosage with HCC risk: relatively small cumulative dosages had comparable or even greater preventive effects, compared with higher dosages.^2–4,8^ Our results related to the recency of statin use may explain these non-linear associations, as smaller-dosage users had lowered risk because they tended to include more recent initiators than their counterparts, although this group did also include early quitters.

As a prospective cohort study, biases related to the retrospective design, such as recall and selection biases, were minimised. Nearly complete follow-up for HCC via linkage to a national database is a further strength. Nonetheless, our study has limitations. Relatively few people received statins, and the cumulative dosages seemed to be relatively low in users. Our study participants were homogeneously Korean, which might affect the generalisability of the study. Additionally, our finding that statin use was associated with a lower risk of HCC when prevalent users were included or cholesterol levels were not adjusted for, as in previous observational studies, enhances the generalisability of our findings.

In conclusion, new statin users experienced no beneficial effects against HCC after adjustment for TC levels measured within 6 months before statin use, whereas TC levels had a strong inverse association with HCC. Observational studies evaluating the effects of statins against HCC should include only new users, after careful consideration of cholesterol levels and other confounders measured just before statin initiation to minimise biases. Overall, our study suggests no protective effect of statins against HCC.

## Supplementary information

Online only supplemental Text, Figure, and Tables

## Data Availability

The data underlying the results presented in the study are available from the National Health Insurance Service (NHIS) (http://nhiss.nhis.or.kr/bd/ab/bdaba000eng.do). Applicants to use the data should contact the NHIS (Office of big data operation, +82-33-736-2469) for further information.
