# Supplementary material for: Higher cholesterol levels, not statin use, are associated with a lower risk of hepatocellular carcinoma
Source: Br J Cancer. 2019 Dec 20;122(5):630–3. doi: 10.1038/s41416-019-0691-3 (PMC7054540; doi:10.1038/s41416-019-0691-3)
Supplement: Supplementary file 1 — Online only supplemental Text, Figure, and Tables [file 41416_2019_691_MOESM1_ESM.pdf]

Online-only Supplementary Data

Higher cholesterol levels, not statin use, are associated with a lower risk of hepatocellular carcinoma

Sang-Wook Yi, Department of Preventive Medicine and Public Health, Catholic Kwandong University College of Medicine, Republic of Korea; **Se Hwa Kim** and **Ki Jun Han**, Department of Internal Medicine, Catholic Kwandong University College of Medicine, Republic of Korea; **Jee-Jeon Yi**, Institute of Epidemiology and Public Health, Catholic Kwandong University, Republic of Korea; **Heechoul Ohrr**, Department of Preventive Medicine, Yonsei University College of Medicine, Republic of Korea

Correspondence: Sang-Wook Yi, MD, PhD, Department of Preventive Medicine and Public Health, Catholic Kwandong University College of Medicine, Bumil-ro 579, Gangneung, Gangwon-do, 25601, Republic of Korea; Tel: +82-33-649-7468; Fax +82-33-641-1074. e-mail: flyhigh@cku.ac.kr

METHODS

Study population

The National Health Insurance Service (NHIS) provides mandatory health insurance for 97% of the Korean population and nationwide biennial health screening.[1, 2] The original cohort (n=514,886) was a 10% random sample of the 5.15 million NHIS beneficiaries aged 40-79 years in 2002 who received a health examination in 2002 or 2003. More details on the national health screening program and original cohort are available elsewhere.[2] Among the cohort, 4669 persons with a history of cancer or missing information were excluded, as were non-participants in health examinations during 2004-2007 (n=91,627) and 208 persons who developed HCC before the 2004-2007 health examinations or were missing information for the 2004-2007 health examination (Figure S1). Since the NHIS prescription database was available during 2002-2013, to assess statin use up to 2 years after (or before) the health examinations, participants in the 2004-2007 examinations were selected for the current analyses. The remaining 418,382 individuals comprised the cohort that included prevalent statin users. The main study cohort (n=400,318) was generated by excluding prevalent statin users during the 6 months before the 2004-2007 health examinations and the first 6 months of follow-up. Additionally, a sensitivity analysis cohort (n=385,945) was created by excluding prevalent users during the 2 years before the 2004-2007 health examinations, to minimize prevalent user bias more thoroughly.

The authors were granted access by the NHIS to the anonymized data. This study was approved by the Institutional Review Board of Catholic Kwandong University, Republic of Korea. Informed consent was waived because the study used anonymized data constructed by the NHIS according to a strong confidentiality protocol.

Study outcomes and follow-up

We followed up study participants from the date of the 2004-2007 baseline health examinations (index date for the analysis) until December 31, 2013 via record linkage to hospital discharge records from the NHIS, in which certified health information managers review the medical records and assign standardized diagnosis codes according to the International Classification of Diseases 10th revision (ICD-10).[3] The completeness of the cancer incidence data from NHIS is comparable to that of the Korea National Cancer Incidence Database, which was estimated to be 97.8%.[4, 5] All patients discharged from the hospital due to HCC (ICD-10: C220) for the first time during follow-up were considered incident cases.

Exposure to statins

Information on the prescriptions of each statin, including atorvastatin, fluvastatin, lovastatin, pitavastatin, pravastatin, rosuvastatin, and simvastatin, was obtained from the NHIS prescription database during 2002-2013. The NHIS prescription database contains prospective records of all prescriptions by doctors to be dispensed by Korean pharmacies. The names of the prescribed drugs, date of prescription, daily

dosage, and duration of each prescription were collected. The defined daily doses (DDDs) recommended by the World Health Organization were used to quantify statin usage.[6] Cumulative DDDs (cDDDs) within 6 months before/after, within 2 years before/after baseline were estimated as the sum of the prescribed DDDs of any statins during the specified period.

Data collection

Total cholesterol (TC) and glucose were assayed using fasting serum samples by enzymatic methods. Alanine aminotransferase (ALT) and aspartate aminotransferase were measured using the NADH-UV method or the Reitman-Frankel method. Systolic blood pressure was measured in a seated position. Body mass index (BMI) was calculated as the measured weight in kilograms divided by the square of measured height in meters (kg/m^2).[1] A single measurement was made at each health examination. Smoking status, alcohol use, physical activity, and history of cancer were assessed via a questionnaire. Health examinations and data collection followed a standard protocol officially documented by the government. The external quality assessments for clinical chemistry in participating hospitals were regularly performed.

Medical risk factors at baseline

We considered individuals to have known prevalent disease at baseline if they visited a medical institution for the diagnosed diseases—either as inpatients or outpatients—at least once within 12 months before or 2 months after the baseline health examination date, via record linkage to healthcare utilization records from the NHIS. The medical risk factors for HCC were selected using the ICD-10 codes: hepatitis B virus (HBV) infection: B16, B180, B181; hepatitis C virus (HCV) infection: B171, B182; diabetes: E10-E14; liver cirrhosis: K74. Persons with fasting glucose level ≥126 mg/dL (7 mmol/L) at the baseline health examinations were also considered to have diabetes.

Statistical analysis

The dosages (cDDDs) and timing of statin use after (or before) baseline were evaluated as continuous and categorical variables. Hazard ratios (HRs) for HCC incidence were calculated using Cox proportional hazards models stratified by age at baseline (years; 40-44, 45-54, 55-64, 65-74, and ≥75) after adjustment for age at baseline (continuous variable; within each age group), sex, smoking status (current smoker, former smoker, never smoker, and missing information [n=17,029]), alcohol use (g ethanol/day; none, <10, 10-39, ≥40, and missing information [n=18,372]), physical activity (at least once a week; yes, and no), beneficiary income status (deciles; below 4 [low income], 4-7, 8-10 [high income]), BMI (continuous variable), ALT (IU/L, <20, 20-39, 40-59, 60-79, and ≥80), diabetes, HBV infection, HCV infection, and liver cirrhosis. Co-variables measured in the 2004-2007 examinations were used in the Cox models, while for the behavioral factors, data collected in the 2002-2003 examinations were used considering the

possibility of behavioral changes due to recent health issues. Analyses with varying cohorts and adjustment for varying confounders were used as sensitivity analyses. All *p* values were 2-sided. All analyses used SAS version 9.4 (SAS Institute Inc., Cary, NC, USA).

REFERENCE

1. Yi SW, Ohrr H, Shin SA, Yi JJ. Sex-age-specific association of body mass index with all-cause mortality among 12.8 million Korean adults: a prospective cohort study. Int J Epidemiol 2015;44:1696-1705.
2. Seong SC, Kim YY, Park SK, et al. Cohort profile: the National Health Insurance Service-National Health Screening Cohort (NHIS-HEALS) in Korea. BMJ Open 2017;7:e016640.
3. Yi SW, Choi JS, Yi JJ, Lee YH, Han KJ. Risk factors for hepatocellular carcinoma by age, sex, and liver disorder status: A prospective cohort study in Korea. Cancer 2018;124:2748-2757.
4. Oh CM, Won YJ, Jung KW, et al. Cancer Statistics in Korea: Incidence, Mortality, Survival, and Prevalence in 2013. Cancer Res Treat 2016;48:436-450.
5. Seo HJ, Oh IH, Yoon SJ. A comparison of the cancer incidence rates between the national cancer registry and insurance claims data in Korea. Asian Pac J Cancer Prev 2012;13:6163-6168.
6. WHO Collaborating Centre for Drug Statistics Methodology. ATC/DDD Index 2018. https://www.whocc.no/atc_ddd_index/, vol. 2018.

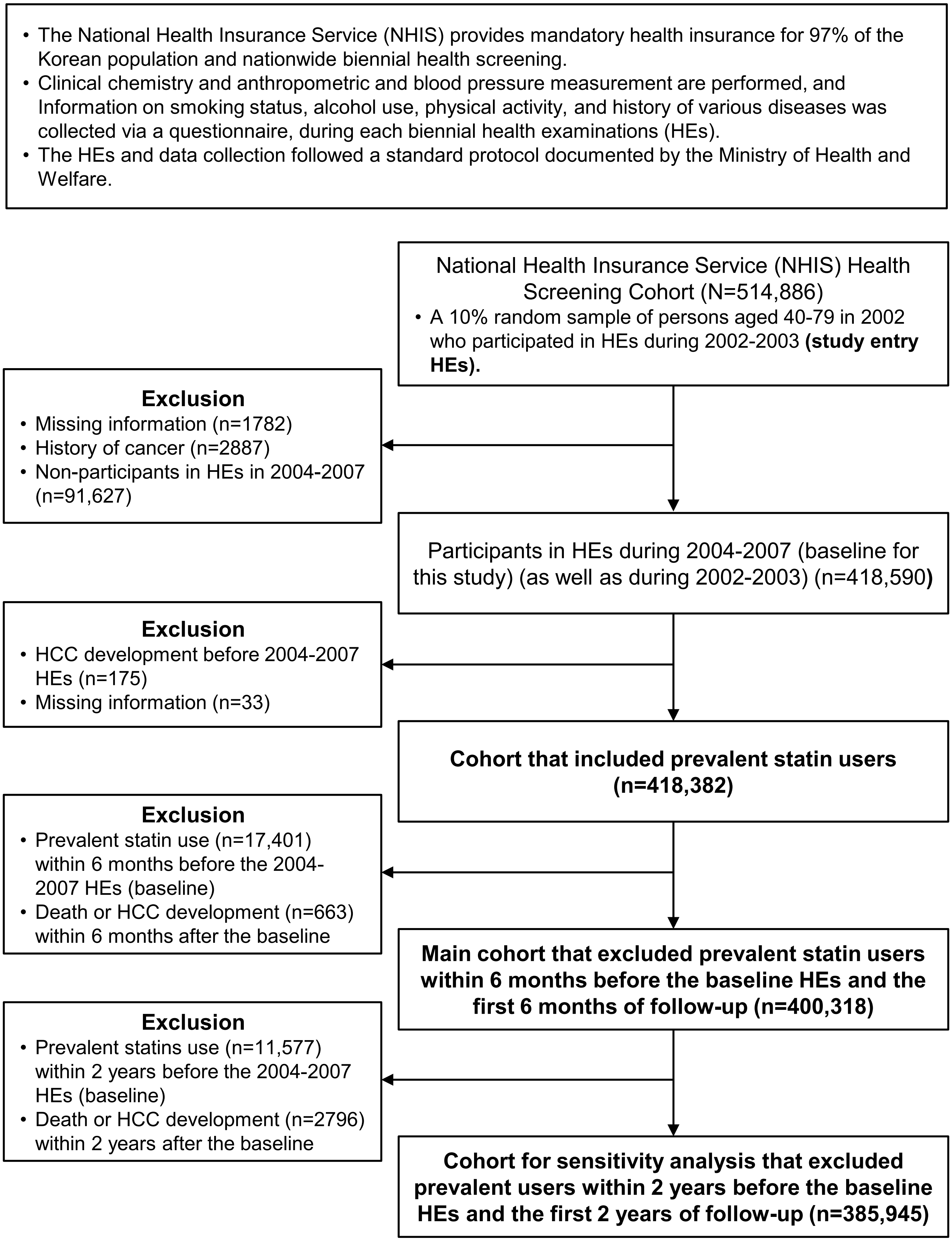

Figure S1. Flow diagram of the study.
HCC, hepatocellular carcinoma; HE, health examination

Table S1. Characteristics of participants according to incident HCC status.

Variables	Characteristics	Total	No HCC	Incident HCC	
		N=400,318	n=398,632	n=1686	*p*
Age	years	54.8 ±9.4	54.8 ±9.4	57.6 ±9.3	<0.001
Total cholesterol[a]	mg/dL	198.6 ±36.7	198.7 ±36.6	174.1 ±35.5	<0.001
Body mass index	kg/m^2	23.9 ±2.9	23.9 ±2.9	23.9 ±3.0	0.619
Sex	Women	178,136 (44.5)	177,864 (44.6)	272 (16.1)	<0.001
	Men	222,182 (55.5)	220,768 (55.4)	1414 (83.9)	
Smoking status	Never smoker	256,473 (64.1)	255,647 (64.1)	826 (49.0)	<0.001
	Past smoker	35,280 (8.8)	35,082 (8.8)	198 (11.7)	
	Current smoker	91,536 (22.9)	90,946 (22.8)	590 (35.0)	
	Missing	17,029 (4.3)	16,957 (4.3)	72 (4.3)	
Alcohol use, g ethanol/day	None	217,990 (54.5)	217,225 (54.5)	765 (45.4)	<0.001
	<10	82,568 (20.6)	82,216 (20.6)	352 (20.9)	
	10-39	72,219 (18.0)	71,845 (18.0)	374 (22.2)	
	≥40	18,372 (4.6)	18,213 (4.6)	159 (9.4)	
	Missing	9,169 (2.3)	9,133 (2.3)	36 (2.1)	
Physical activity	≥1 times/week	168,723 (42.1)	168,003 (42.1)	720 (42.7)	0.642
Income status, decile	<4 (low-income)	89,060 (22.2)	88,630 (22.2)	430 (25.5)	0.003
	4-7	128,794 (32.2)	128,253 (32.2)	541 (32.1)	
	>7 (high-income)	182,464 (45.6)	181,749 (45.6)	715 (42.4)	
Alanine aminotransferase,	<20 IU/L	176,623 (44.1)	176,462 (44.3)	161 (9.5)	<0.001
	20-39 IU/L	178,698 (44.6)	178,067 (44.7)	631 (37.4)	
	40-59 IU/L	30,845 (7.7)	30,429 (7.6)	416 (24.7)	
	60-79 IU/L	8,059 (2.0)	7,845 (2.0)	214 (12.7)	
	≥80 IU/L	6,093 (1.5)	5,829 (1.5)	264 (15.7)	
Diabetes[b]	Yes	44,059 (11.0)	43,700 (11.0)	359 (21.3)	<0.001
Prevalent liver disease	HBV infection	3,499 (0.9)	3,221 (0.8)	278 (16.5)	<0.001
	HCV infection	669 (0.2)	605 (0.2)	64 (3.8)	<0.001
	Liver cirrhosis	1,305 (0.3)	1,075 (0.3)	230 (13.6)	<0.001
Statin use during 6 months	No use	389,052 (97.2)	387,395 (97.2)	1657 (98.3)	0.006
after baseline	Use	11,266 (2.8)	11,237 (2.8)	29 (1.7)	
cDDDs during	No use	389,052 (97.2)	387,395 (97.2)	1657 (98.3)	0.025
6 months after baseline	≤91	9,350 (2.3)	9,326 (2.3)	24 (1.4)	
	>91	1,916 (0.5)	1,911 (0.5)	5 (0.3)	
Statin use within 2 years	No use	366,896 (91.7)	365,276 (91.6)	1620 (96.1)	<0.001
after baseline	Use	33,422 (8.3)	33,356 (8.4)	66 (3.9)	

cDDDs within	No use	366,896	(91.7)	365,276	(91.6)	1620	(96.1)	<0.001
2 years after baseline	≤182 days	25,288	(6.3)	25,237	(6.3)	51	(3.0)	
	182.1-365 days	5,741	(1.4)	5,733	(1.4)	8	(0.5)	
	>365 days	2,393	(0.6)	2,386	(0.6)	7	(0.4)	
Timing of statin initiation	No use	366,896	(91.7)	365,276	(91.6)	1620	(96.1)	<0.001
after baseline	≤182 days	11,266	(2.8)	11,237	(2.8)	29	(1.7)	
	183-365 days	7,085	(1.8)	7,070	(1.8)	15	(0.9)	
	>365 days	15,071	(3.8)	15,049	(3.8)	22	(1.3)	

cDDDs, cumulative defined daily doses; HCC, hepatocellular carcinoma; HBV, hepatitis B virus; HCV, hepatitis C virus.
Data are expressed as mean±SD or n (%).
The *p* values were calculated by the chi-square test and 1-way ANOVA between HCC development status.
[a]To convert total cholesterol from mg/dL to mmol/L, multiply by 0.02586.
[b]Known prevalent diabetes or fasting glucose ≥126 mg/dL at baseline.

Table S2. Characteristics of participants according to statin use within 6 months after baseline health examinations.

Variables	Characteristics	Total	Non user	Statin user	
		N=400,318	n=389,052	n=11,266	*p*
Age	years	54.8 ±9.4	54.7 ±9.4	58.0 ±8.8	<0.001
Total cholesterol[a]	mg/dL	198.6 ±36.7	197.2 ±35.5	247.8 ±42.7	<0.001
Body mass index	kg/m^2	23.9 ±2.9	23.9 ±2.9	24.8 ±2.9	<0.001
Sex	Women	178,136 (44.5)	171,749 (44.1)	6,387 (56.7)	<0.001
	Men	222,182 (55.5)	217,303 (55.9)	4,879 (43.3)	
Smoking status	Never smoker	256,473 (64.1)	248,583 (63.9)	7,890 (70.0)	<0.001
	Past smoker	35,280 (8.8)	34,431 (8.8)	849 (7.5)	
	Current smoker	91,536 (22.9)	89,487 (23.0)	2,049 (18.2)	
	Missing	17,029 (4.3)	16,551 (4.3)	478 (4.2)	
Alcohol use, g ethanol/day	None	217,990 (54.5)	211,021 (54.2)	6,969 (61.9)	<0.001
	<10	82,568 (20.6)	80,685 (20.7)	1,883 (16.7)	
	10-39	72,219 (18.0)	70,565 (18.1)	1,654 (14.7)	
	≥40	18,372 (4.6)	17,920 (4.6)	452 (4.0)	
	Missing	9,169 (2.3)	8,861 (2.3)	308 (2.7)	
Physical activity	≥1 times/week	168,723 (42.1)	163,913 (42.1)	4,810 (42.7)	0.232
Income status, decile	<4 (low-income)	89,060 (22.2)	86,725 (22.3)	2,335 (20.7)	<0.001
	4-7	128,794 (32.2)	125,134 (32.2)	3,660 (32.5)	
	>7 (high-income)	182,464 (45.6)	177,193 (45.5)	5,271 (46.8)	
Alanine aminotransferase,	<20 IU/L	176,623 (44.1)	172,719 (44.4)	3,904 (34.7)	<0.001
	20-39 IU/L	178,698 (44.6)	173,216 (44.5)	5,482 (48.7)	
	40-59 IU/L	30,845 (7.7)	29,629 (7.6)	1,216 (10.8)	
	60-79 IU/L	8,059 (2.0)	7,680 (2.0)	379 (3.4)	
	≥80 IU/L	6,093 (1.5)	5,808 (1.5)	285 (2.5)	
Diabetes[b]	Yes	44,059 (11.0)	41,610 (10.7)	2,449 (21.7)	<0.001
Incident HCC during follow-up	Yes	1,686 (0.42)	1657 (0.43)	29 (0.26)	0.006
Prevalent liver disease	HBV infection	3,499 (0.9)	3,427 (0.9)	72 (0.6)	0.007
	HCV infection	669 (0.2)	659 (0.2)	10 (0.1)	0.039
	Liver cirrhosis	1,305 (0.3)	1,294 (0.3)	11 (0.1)	<0.001
Statin use during 6 months	No use	389,052 (97.2)	389,052 (100)	0 (0.0)	<0.001
after baseline	Use	11,266 (2.8)	0 (0.0)	11,266 (100)	
cDDDs during	No use	389,052 (97.2)	389,052 (100)	0 (0.0)	<0.001
6 months after baseline	≤91	9,350 (2.3)	0 (0.0)	9,350 (83.0)	
	>91	1,916 (0.5)	0 (0.0)	1,916 (17.0)	

Statin use within 2 years	No use	366,896 (91.7)	366,896 (94.3)	0 (0.0)	<0.001
after baseline	Use	33,422 (8.3)	22,156 (5.7)	11,266 (100)	
cDDDs within	No use	366,896 (91.7)	366,896 (94.3)	0 (0.0)	<0.001
2 years after baseline	≤182 days	25,288 (6.3)	18,304 (4.7)	6,984 (62.0)	
	182.1-365 days	5,741 (1.4)	3,133 (0.8)	2,608 (23.1)	
	>365 days	2,393 (0.6)	719 (0.2)	1,674 (14.9)	
Timing of statin initiation	No use	366,896 (91.7)	366,896 (94.3)	0 (0.0)	<0.001
after baseline	≤182 days	11,266 (2.8)	0 (0.0)	11,266 (100)	
	183-365 days	7,085 (1.8)	7,085 (1.8)	0 (0.0)	
	>365 days	15,071 (3.8)	15,071 (3.9)	0 (0.0)	

cDDDs, cumulative defined daily doses; HCC, hepatocellular carcinoma; HBV, hepatitis B virus; HCV, hepatitis C virus.
Data are expressed as mean±SD or n (%).
The *p* values were calculated by the chi-square test and 1-way ANOVA between HCC development status.
[a]To convert total cholesterol from mg/dL to mmol/L, multiply by 0.02586.
[b]Known prevalent diabetes or fasting glucose ≥126 mg/dL at baseline.

Table S3. HRs[a] for HCC incidence associated with statin use and total cholesterol in prevalent user included cohort (n=418,382)

TC or statins use characteristics	No. of participants	HCC cases	Age-sex-adjusted *p*-value	Age-sex-adjusted HR (95% CI)	Multivariable-except TC-adjusted *p*-value	Multivariable-except TC-adjusted HR (95% CI)	Multivariable-including TC-adjusted (Main analysis) *p*-value	Multivariable-including TC-adjusted (Main analysis) HR (95% CI)
1 mmol/L (39 mg/dL) increase in TC[b]	418,382	1842	<0.001	0.47 (0.44-0.49)			<0.001	0.56 (0.53-0.59)
Statin use within past 6 months before baseline								
No use	400,981	1814		1.00 (Reference)		1.00 (Reference)		1.00 (Reference)
Use	17,401	28	<0.001	0.35 (0.24-0.51)	<0.001	0.31 (0.22-0.46)	<0.001	0.27 (0.18-0.39)
≤91 cDDDs	12,502	19	<0.001	0.33 (0.21-0.52)	<0.001	0.30 (0.19-0.46)	<0.001	0.28 (0.18-0.44)
>91 cDDDs	4,899	9	0.005	0.39 (0.20-0.75)	0.002	0.36 (0.19-0.69)	<0.001	0.25 (0.13-0.47)
per 60 cDDD increase	418,382	1842	<0.001	0.53 (0.39-0.70)	<0.001	0.48 (0.36-0.65)	<0.001	0.42 (0.32-0.57)
Statin use within past 2 years before baseline								
No use	389,291	1786		1.00 (Reference)		1.00 (Reference)		1.00 (Reference)
Use	29,091	56	<0.001	0.42 (0.32-0.54)	<0.001	0.38 (0.29-0.49)	<0.001	0.39 (0.30-0.51)
≤182 cDDDs	21,315	40	<0.001	0.41 (0.30-0.56)	<0.001	0.37 (0.27-0.51)	<0.001	0.43 (0.32-0.59)
183-365 cDDDs	5,437	10	0.002	0.38 (0.20-0.71)	<0.001	0.34 (0.18-0.64)	<0.001	0.29 (0.16-0.54)
>365 cDDDs	2,339	6	0.127	0.54 (0.24-1.19)	0.098	0.51 (0.23-1.13)	0.013	0.36 (0.16-0.80)
per 60 cDDD increase	418,382	1842	<0.001	0.81 (0.73-0.89)	<0.001	0.78 (0.71-0.87)	<0.001	0.77 (0.70-0.85)
No use	389,291	1786		1.00 (Reference)		1.00 (Reference)		1.00 (Reference)
Last prescription ≤182 days before baseline (Prevalent users within 6 months before baseline)	17,401	28	<0.001	0.34 (0.24-0.50)	<0.001	0.31 (0.21-0.45)	<0.001	0.27 (0.18-0.39)
Last prescription 183-365 days before baseline	4,821	7	0.003	0.32 (0.15-0.68)	0.002	0.32 (0.15-0.66)	0.045	0.47 (0.22-0.98)
Last prescription 366-730 days before baseline	6,869	21	0.068	0.67 (0.44-1.03)	0.017	0.59 (0.38-0.91)	0.547	0.88 (0.57-1.35)

			Age-sex-adjusted		Multivariable-except TC-adjusted		Multivariable-including TC -adjusted (Main analysis)	
TC or statins use characteristics	No. of participants	HCC cases	*p*-value	HR (95% CI)	*p*-value	HR (95% CI)	*p*-value	HR (95% CI)
1 mmol/L (39 mg/dL) increase in TC[c]	418,382	1842	<0.001	0.47 (0.45-0.50)			<0.001	0.56 (0.53-0.59)
Statin use within 6 months after baseline								
No use	394,133	1793		1.00 (Reference)		1.00 (Reference)		1.00 (Reference)
Use	24,249	49	<0.001	0.45 (0.34-0.60)	<0.001	0.41 (0.31-0.54)	<0.001	0.45 (0.34-0.60)
≤91 cDDDs	17,073	33	<0.001	0.44 (0.31-0.61)	<0.001	0.40 (0.28-0.56)	<0.001	0.49 (0.35-0.69)
>91 cDDDs	7,176	16	0.004	0.48 (0.29-0.79)	<0.001	0.43 (0.26-0.71)	<0.001	0.38 (0.23-0.62)
per 60 cDDD increase	418,382	1842	<0.001	0.56 (0.45-0.71)	<0.001	0.53 (0.42-0.67)	<0.001	0.55 (0.44-0.68)
Statin use within 2 years after baseline								
No use	370,250	1754		1.00 (Reference)		1.00 (Reference)		1.00 (Reference)
Use	48,132	88	<0.001	0.39 (0.31-0.48)	<0.001	0.37 (0.29-0.45)	<0.001	0.45 (0.36-0.56)
≤182 cDDDs	30,220	58	<0.001	0.42 (0.32-0.54)	<0.001	0.40 (0.30-0.52)	<0.001	0.55 (0.42-0.71)
183-365 cDDDs	10,670	10	<0.001	0.20 (0.10-0.36)	<0.001	0.18 (0.10-0.33)	<0.001	0.21 (0.11-0.38)
>365 cDDDs	7,242	20	0.006	0.54 (0.35-0.84)	0.004	0.52 (0.33-0.81)	0.001	0.48 (0.31-0.74)
per 60 cDDD increase	418,382	1842	<0.001	0.83 (0.78-0.88)	<0.001	0.82 (0.77-0.87)	<0.001	0.84 (0.80-0.89)

cDDDs cumulative DDDs; CI, confidence interval; DDD, defined daily dose; HCC, hepatocellular carcinoma; HR, hazard ratio; TC, total cholesterol

[a] HRs were calculated by Cox models stratified by age (baseline age, years: 40-44, 45-54, 55-64, 65-74, ≥75), after adjustment for age at baseline, sex, pre-existing diabetes, smoking status, alcohol use, physical activity, hepatitis B virus infection, hepatitis C virus infection, liver cirrhosis, body mass index, and alanine aminotransferase.

[b] Statins use within past 6 months before baseline was used in the multivariable analysis.

[c] Statins use within 6 months after baseline was used in the multivariable analysis.

To convert cholesterol from mg/dL to mmol/L, multiply by 0.02586

Table S4. HRs[a] for HCC incidence associated with statin use and total cholesterol (TC) according to TC levels at baseline in main cohort participants (n=400,318, Sensitivity analysis)

TC, mg/dL	TC or statins use characteristics	No. of participants	HCC cases	Multivariable-except TC-adjusted		Multivariable-including TC-adjusted	
				p-value	HR (95% CI)	*p*-value	HR (95% CI)
All participants	1 mmol/L (39 mg/dL) increase in TC	400,318	1686			<0.001	0.54 (0.51-0.58)
	Statin use within 6 months after baseline						
	No use	389,052	1,657		1.00 (Reference)		1.00 (Reference)
	Use	11,266	29	0.005	0.59 (0.41-0.85)	0.427	1.16 (0.80-1.69)
240≤TC<400 mg/dL	1 mmol/L (39 mg/dL) increase in TC	51,118	63			0.283	0.77 (0.48-1.24)
	Statin use within 6 months after baseline						
	No use	44,296	55		1.00 (Reference)		1.00 (Reference)
	Use	6,822	8	0.816	0.91 (0.43-1.94)	0.952	0.98 (0.46-2.09)
200≤TC<240 mg/dL	1 mmol/L (39 mg/dL) increase in TC	133,509	286			0.166	0.75 (0.50-1.13)
	Statin use within 6 months after baseline						
	No use	130,552	278				1.00 (Reference)
	Use	2,957	8	0.958	0.98 (0.48-1.99)	0.966	1.02 (0.50-2.07)
TC<200 mg/dL	1 mmol/L (39 mg/dL) increase in TC	215,587	1336			<0.001	0.57 (0.52-0.63)
	Statin use within 6 months after baseline						
	No use	214,131	1,323		1.00 (Reference)		1.00 (Reference)
	Use	1,456	13	0.432	1.25 (0.72-2.15)	0.223	1.41 (0.81-2.43)

HCC, hepatocellular carcinoma; HR, hazard ratio; TC, total cholesterol

[a] HRs were calculated by Cox models stratified by age (baseline age, years: 40-44, 45-54, 55-64, 65-74, ≥75), after adjustment for age at baseline, sex, pre-existing diabetes, smoking status, alcohol use, physical activity, hepatitis B virus infection, hepatitis C virus infection, liver cirrhosis, body mass index, and alanine aminotransferase.

To convert cholesterol from mg/dL to mmol/L, multiply by 0.02586.

Table S5. HRs[a] for HCC incidence associated with statin use and total cholesterol (TC) after excluding prevalent users within 2 years before baseline and first 2 years of follow-up (n=385,945, Sensitivity analysis)

			Age-sex-adjusted		Multivariable-except TC-adjusted		Multivariable-including TC- adjusted (Main analysis)	
TC or statins use characteristics	No. of participants	HCC cases	*p*-value	HR (95% CI)	*p*-value	HR (95% CI)	*p*-value	HR (95% CI)
1 mmol/L (39 mg/dL) increase in TC[b]	385,945	1363	<0.001	0.46 (0.43-0.50)			<0.001	0.56 (0.52-0.59)
Statin use within 6 months after baseline								
No use	376,966	1345		1.00 (Reference)		1.00 (Reference)		1.00 (Reference)
Use	8,979	18	0.031	0.60 (0.38-0.95)	0.010	0.54 (0.34-0.86)	0.948	1.02 (0.63-1.63)
≤91 cDDDs	7,500	14	0.028	0.55 (0.33-0.94)	0.008	0.49 (0.29-0.83)	0.728	0.91 (0.53-1.55)
>91 cDDDs	1,479	4	0.727	0.84 (0.31-2.24)	0.744	0.85 (0.32-2.27)	0.282	1.72 (0.64-4.60)
per 60 cDDD increase	385,945	1363	0.159	0.74 (0.49-1.12)	0.094	0.69 (0.45-1.06)	0.560	1.12 (0.77-1.62)
<40 cDDDs (<50th percentile)	3,951	9	0.223	0.66 (0.35-1.28)	0.111	0.59 (0.30-1.13)	0.898	1.04 (0.54-2.02)
≥40 cDDDs (≥50th percentile)	5,028	9	0.069	0.54 (0.28-1.05)	0.039	0.50 (0.26-0.96)	0.974	0.99 (0.51-1.91)
Statin use within 2 years after baseline								
No use	357,465	1316		1.00 (Reference)		1.00 (Reference)		1.00 (Reference)
Use	28,480	47	<0.001	0.46 (0.34-0.61)	<0.001	0.44 (0.33-0.59)	0.007	0.67 (0.50-0.90)
≤182 cDDDs	21,917	33	<0.001	0.43 (0.30-0.60)	<0.001	0.41 (0.29-0.58)	0.006	0.61 (0.43-0.87)
183-365 cDDDs	4,616	7	0.018	0.41 (0.19-0.86)	0.011	0.38 (0.18-0.80)	0.156	0.58 (0.28-1.23)
>365 cDDDs	1,947	7	0.840	0.93 (0.44-1.95)	0.780	0.90 (0.43-1.89)	0.286	1.50 (0.71-3.17)
per 60 cDDD increase	385,945	1363	0.004	0.87 (0.79-0.95)	0.002	0.85 (0.77-0.94)	0.336	0.96 (0.87-1.05)
<30 cDDDs (1st quartile)	7,083	14	0.026	0.55 (0.32-0.93)	0.020	0.53 (0.31-0.90)	0.369	0.78 (0.46-1.33)
30-79 cDDDs (2nd quartile)	7,482	8	<0.001	0.30 (0.15-0.61)	<0.001	0.30 (0.15-0.60)	0.021	0.44 (0.22-0.88)

80-179 cDDDs (3rd quartile)	7,003	10	0.005	0.41 (0.22-0.76)	0.002	0.37 (0.20-0.70)	0.103	0.59 (0.32-1.11)
≥180 cDDDs (4th quartile)	6,912	15	0.035	0.58 (0.35-0.96)	0.019	0.54 (0.33-0.91)	0.544	0.85 (0.51-1.43)
Statin initiation ≤182 days after baseline	8,979	18	0.021	0.58 (0.36-0.92)	0.006	0.52 (0.32-0.82)	0.934	0.98 (0.61-1.57)
Statin initiation 183-365 days after baseline	5,997	11	0.023	0.50 (0.28-0.91)	0.013	0.47 (0.26-0.85)	0.209	0.68 (0.38-1.24)
Statin initiation >365 days after baseline	13,504	18	<0.001	0.37 (0.23-0.58)	<0.001	0.37 (0.23-0.58)	0.004	0.50 (0.31-0.80)

cDDDs cumulative DDDs; CI, confidence interval; DDD, defined daily dose; HCC, hepatocellular carcinoma; HR, hazard ratio; TC, total cholesterol

[a] HRs were calculated by Cox models stratified by age (baseline age, years: 40-44, 45-54, 55-64, 65-74, ≥75), after adjustment for age at baseline, sex, pre-existing diabetes, smoking status, alcohol use, physical activity, hepatitis B virus infection, hepatitis C virus infection, liver cirrhosis, body mass index, and systolic blood pressure.

[b] Statins use within 6 months after baseline was used in the multivariable analysis.

To convert cholesterol from mg/dL to mmol/L, multiply by 0.02586.

Table S6. HRs[a] for HCC incidence associated with statin use and total cholesterol (TC) according to liver diseases status in the main cohort (n=400,318) and prevalent user included cohort (n=418,382)

					Multivariable-except TC-adjusted		Multivariable-including TC -adjusted (Main model)	
		TC or statins use characteristics	No. of participants	HCC cases	*p*-value	HR (95% CI)	*p*-value	HR (95% CI)
**Main Cohort n=400318**	Any LD defined by ALT≥40 or AST≥40 or diagnostic codes for viral hepatitis or LD[b]	1 mmol/L (39 mg/dL) increase in TC	70,179	1269			<0.001	0.55 (0.52-0.59)
		Statin use within 6 months after baseline						
		No use	67,410	1,250		1.00 (Reference)		1.00 (Reference)
		Use	2,769	19	<0.001	0.47 (0.30-0.73)	0.730	0.92 (0.58-1.46)
	No LD defined by ALT<40 and AST<40 and no diagnostic codes for viral hepatitis or LD[b]	1 mmol/L (39 mg/dL) increase in TC	330,139	417			<0.001	0.55 (0.49-0.62)
		Statin use within 6 months after baseline						
		No use	321,642	407		1.00 (Reference)		1.00 (Reference)
		Use	8,497	10	0.694	0.88 (0.47-1.65)	0.162	1.58 (0.83-2.98)
	LD defined by diagnostic codes for viral hepatitis or LD[b]	1 mmol/L (39 mg/dL) increase in TC	23,690	677			<0.001	0.60 (0.54-0.65)
		Statin use within 6 months after baseline						
		No use	22,668	667		1.00 (Reference)		1.00 (Reference)
		Use	1,022	10	0.019	0.47 (0.25-0.88)	0.734	0.90 (0.47-1.69)
	No LD defined by no diagnostic codes for viral hepatitis or LD[b]	1 mmol/L (39 mg/dL) increase in TC	376,628	1009			<0.001	0.53 (0.49-0.57)
		Statin use within 6 months after baseline						
		No use	366,384	990		1.00 (Reference)		1.00 (Reference)
		Use	10,244	19	0.028	0.60 (0.38-0.95)	0.507	1.17 (0.74-1.85)

					Multivariable-except TC-adjusted		Multivariable-including TC -adjusted (Main model)	
		TC or statins use characteristics	No. of participants	HCC cases	*p*-value	HR (95% CI)	*p*-value	HR (95% CI)
**Prevalent**	Any LD defined by	1 mmol/L (39 mg/dL) increase in TC	75,013	1391			<0.001	0.58 (0.55-0.62)
**User**	ALT≥40 or AST≥40 or	***Statin use within 6 months after baseline***						
**Included**	diagnostic codes for	No use	68,931	1,364		1.00 (Reference)		1.00 (Reference)
**Cohort**	viral hepatitis or LD[b]	Use	6,082	27	<0.001	0.27 (0.19-0.40)	<0.001	0.31 (0.21-0.46)
**n=418382**	No LD defined by	1 mmol/L (39 mg/dL) increase in TC	343,369	451			<0.001	0.58 (0.52-0.64)
	ALT<40 and AST<40 and	***Statin use within 6 months after baseline***						
	no diagnostic codes for	No use	325,202	429		1.00 (Reference)		1.00 (Reference)
	viral hepatitis or LD[b]	Use	18,167	22	0.222	0.76 (0.49-1.18)	0.240	0.77 (0.50-1.19)
	LD defined by	1 mmol/L (39 mg/dL) increase in TC	25,792	763			<0.001	0.63 (0.57-0.68)
	diagnostic codes for	***Statin use within 6 months after baseline***						
	viral hepatitis or LD[b]	No use	23,512	750		1.00 (Reference)		1.00 (Reference)
		Use	2,280	13	<0.001	0.25 (0.14-0.43)	<0.001	0.31 (0.18-0.53)
	No LD defined by	1 mmol/L (39 mg/dL) increase in TC	392,590	1079			<0.001	0.55 (0.51-0.59)
	no diagnostic codes for	***Statin use within 6 months after baseline***						
	viral hepatitis or LD[b]	No use	370,621	1,043		1.00 (Reference)		1.00 (Reference)
		Use	21,969	36	<0.001	0.55 (0.39-0.77)	<0.001	0.57 (0.40-0.79)

ALT, alanine aminotransferase; CI, confidence interval; HCC, hepatocellular carcinoma; HR, hazard ratio; LD, liver diseases; TC, total cholesterol

[a] HRs were calculated by Cox models stratified by age (baseline age, years: 40-44, 45-54, 55-64, 65-74, ≥75), after adjustment for age at baseline, sex, pre-existing diabetes, smoking status, alcohol use, physical activity, hepatitis B virus infection (when applicable), hepatitis C virus infection (when applicable), liver cirrhosis (when applicable), body mass index, and ALT (when applicable).

[b] Individuals were considered to have viral hepatitis (B15-B19) or non-viral liver diseases (K70-K77) at baseline if they visited a medical institution for the diseases - either as inpatients or outpatients - at least once within 12 months before or 2 months after the baseline health examination date.

To convert cholesterol from mg/dL to mmol/L, multiply by 0.02586.

Table S7. Total cholesterol (TC) levels at health examinations (HEs) during 2002-2003 and 2004-2007 according to statin use status around the time of the HEs in prevalent user included cohort (n=418,382)

	New users or re-initiators[b]		Statin quitters at the time of 2004-2007 HEs[c]	Statin-unaffected TC measurements available[d]		Sex and age-adjusted TC-related measurements[e], mg/dL					Statin use around the time of the 2002-2003 HEs		Statin use around the time of the 2004-2007 HEs	
Statin use status group[a]	After 2002-2003 HEs	After 2004-2007 HEs		At 2002-2003 HEs	At 2004-2007 HEs	No. of participants	Mean TC levels at 2002-2003 HEs (95% CIs)[f]	Mean TC levels at 2004-2007 HEs (95% CIs)[f]	Sex/age-adjusted TC difference between HEs	P value for TC difference	Within 6 months before	Within 6 months after	Within 6 months before	Within 6 months after
1				Yes	Yes	383,168	**197.8 (197.7-197.9)**	**196.8 (196.6-196.9)**	-1.1	<0.001	No	No	No	No
2		Yes		Yes	Yes	9750	**230.3 (229.6-231.1)**	**245.7 (245.0-246.4)**	15.4	<0.001	No	No	No	Yes
3				Yes		3377	**226.1 (224.8-227.3)**	208.9 (207.7-210.1)	-17.2	<0.001	No	No	Yes	No
4				Yes		7396	**228.4 (227.5-229.2)**	186.8 (186.0-187.6)	-41.6	<0.001	No	No	Yes	Yes
5	Yes		Yes	Yes	Yes	3397	**246.7 (245.5-248.0)**	**224.5 (223.3-225.7)**	-22.3	<0.001	No	Yes	No	No
6	Yes	Yes	Yes	Yes	Yes	790	**265.3 (262.7-267.9)**	**257.1 (254.6-259.6)**	-8.3	<0.001	No	Yes	No	Yes
7	Yes			Yes		555	**260.1 (257.0-263.2)**	220.1 (217.2-223.1)	-39.9	<0.001	No	Yes	Yes	No
8	Yes			Yes		1974	**252.7 (251.0-254.3)**	197.0 (195.4-198.6)	-55.7	<0.001	No	Yes	Yes	Yes
9			Yes		Yes	1807	216.0 (214.2-217.7)	**222.1 (220.4-223.7)**	6.1	<0.001	Yes	No	No	No
10		Yes	Yes		Yes	330	230.0 (226.0-234.0)	**254.6 (250.7-258.5)**	24.6	<0.001	Yes	No	No	Yes
11						137	222.9 (216.7-229.1)	221.6 (215.6-227.6)	-1.4	0.647	Yes	No	Yes	No
12						464	222.7 (219.3-226.1)	207.7 (204.4-210.9)	-15.0	<0.001	Yes	No	Yes	Yes
13			Yes		Yes	1330	201.9 (199.9-203.9)	**221.0 (219.1-222.9)**	19.1	<0.001	Yes	Yes	No	No
14		Yes	Yes		Yes	409	216.9 (213.3-220.5)	**251.8 (248.4-255.3)**	34.9	<0.001	Yes	Yes	No	Yes
15						362	212.0 (208.2-215.8)	213.2 (209.5-216.9)	1.2	0.509	Yes	Yes	Yes	No
16						3136	204.0 (202.7-205.3)	193.7 (192.4-194.9)	-10.3	<0.001	Yes	Yes	Yes	Yes

[a] Statin use status was classified into 16 groups according to statin use within 6 months before/after 2002-2003 HEs and within 6 months before/after 2004-2007 HEs.
[b] New users (or re-initiators) were defined as those who had used no statins within 6 months before HEs but started using within 6 months after HEs.
[c] Statin quitters at the time of 2004-2007 HEs were defined as those who had used statin around the time of the 2002-2003 HEs but quit statins at least 6 months before 2004-2007 HEs.
[d] Statin-unaffected TC measurements were defined as TC levels in those who had used no statin within 6 months before HEs.
[e] The mean of TC levels (95% Cis) and the differences in TC levels between HEs were the estimated sex and age adjusted marginal means calculated by LSMEANS statements in generalized linear models using SAS 9.4
[f] Statin-unaffected TC measurements were highlighted with bold font

To convert cholesterol from mg/dL to mmol/L, multiply by 0.02586.
